# Preference for producer specific exudates shapes microbial communities in coral reefs

**DOI:** 10.7717/peerj.20748

**Published:** 2026-02-09

**Authors:** Milou G.I. Arts, Benjamin Mueller, Linda Wegley Kelly, Craig E. Nelson, Irina Koester, Daniel Petras, Ellen Hopmans, Mark J.A. Vermeij, Andreas F. Haas

**Affiliations:** 1 Marine Microbiology and Biogeochemistry, Royal Netherlands Institute for Sea Research, t’Horntje (Texel), Netherlands; 2 Department of Marine Ecology, Universität Bremen, Bremen, Germany; 3 Department of Freshwater and Marine Ecology, University of Amsterdam, Amsterdam, Netherlands; 4 CARMABI, Willemstad, Curacao; 5 Scripps Institution of Oceanography, San Diego, CA, United States of America; 6 Daniel K. Inouye Center for Microbial Oceanography: Research and Education, Department of Oceanography and Sea Grant College Program, University of Hawai‘i at Mānoa, Honolulu, HI, United States of America; 7 Department of Biochemistry, University of California Riverside, Riverside, CA, United States of America

**Keywords:** Coral Reef, Exometabolome, Microbial reminealization

## Abstract

Exometabolites released by benthic primary producers (BPP) are an integral part of the coral reef food web. Depending on their origin and composition these complex mixtures of dissolved organic compounds support a distinct microbial community. Which exudate components are preferentially used by microbes, how this preference differs between exudate types, and what molecular features drive the microbial community differentiation is still poorly understood. Here we use an untargeted metabolomics approach (liquid chromatography-tandem mass spectrometry (LC/MS-MS)) to assess the microbial uptake of exudates produced by BPP (mixed coral community, macroalgae, turf algae, and coral-macroalgae, coral-turf algae). We can show that that exudate compounds and especially those unique to a specific BPP or mixed community are the most favored substrate for microbes in the respective communities. Our data suggests that in each BPP treatment the unique combination of organic compounds is the main driver selecting for a specific microbial community composition rather than a specific single substance. This emphasizes the complexity of mechanisms and metabolisms that constitute and structure communities in ecosystems as intricate as coral reefs.

## Introduction

Dissolved organic matter (DOM) plays a key role in global elemental cycles (Björkman & Karl, 2001; Hansell & Carlson, 2001; Sipler & Bronk, 2015) and the overall functioning of terrestrial and aquatic ecosystems (Kalbitz et al., 2000; Thomas, 1997). The transformation of DOM into bacterial biomass and its subsequent consumption by higher trophic levels forms the foundation of marine food webs (Azam et al., 1983; Azam & Malfatti, 2007). DOM re-cycling appears to be especially important in the functioning of tropical coral reefs (Nelson, Wegley Kelly & Haas, 2023; Silveira et al., 2017; Wild et al., 2004). In contrast to the open ocean, the local DOM pool in these tropical coral reef ecosystems is often largely fueled by an abundant benthic primary producer (BPP) community (Tanaka et al., 2011; Torréton et al., 1997; van Duyl & Gast, 2001). The rapid transformation of benthic-derived DOM into organic particles and the subsequent transfer to higher trophic levels *via* the microbial loop (Azam et al., 1983) and the sponge loop (De Goeij et al., 2013) are suggested to reduce the loss of autochthonously produced energy and nutrients to the open ocean. Thus, DOM re-cycling appears to be a cornerstone that allowed coral reefs to thrive as the most productive and diverse marine ecosystems despite being surrounded by a nutrient-poor ocean (Hatcher, 1988; Odum & Odum, 1955).

These efficient DOM re-cycling pathways that initially allowed coral reefs to evolve under oligotrophic conditions, are now hypothesized to reinforce ongoing benthic community shifts caused by a combination of global (*e.g.*, global warming, ocean acidification) and local stressors (*e.g.*, eutrophication, overfishing) (De Goeij, Lesser & Pawlik, 2017; Dinsdale & Rohwer, 2008; Haas et al., 2016; Pawlik, Burkepile & Thurber, 2016). Particularly in the Caribbean so-called phase shifts from corals and other calcifying taxa to fleshy and turfing algae (Reverter et al., 2022; Smith et al., 2016; Tebbett, Connolly & Bellwood, 2023) are considered to significantly alter DOM dynamics. Firstly, algae typically release more DOM per surface area than corals (Haas et al., 2011; Mueller et al., 2014) which may lead to an increase in benthic DOM production. And secondly, the distinct composition of algal-DOM was reported to stimulate microbial respiration and to fuel the fast, but inefficient growth of opportunistic microbes (*i.e.,* low conversion rate of DOM into bacterial biomass) (Haas et al., 2011; Mueller et al., 2022; Nelson et al., 2013; Thobor et al., 2024). This combined change in DOM quantity and quality results in the depletion of the local DOM pool to sustain a greater microbial biomass and simultaneously reducing the transfer of energy and nutrients to higher trophic levels—the microbialization of reefs (Haas et al., 2016; McDole et al., 2012). At coral–algal interfaces, algal-derived DOM stimulates the growth of opportunistic and potentially pathogenic microbes, which may contribute to coral diseases (Barott & Rohwer, 2012). In combination with prolonged periods of hypoxia (Candy et al., 2023) caused by algal-DOM induced microbial respiration (Kline et al., 2006; Roach et al., 2020; Smith et al., 2006), this can harm or even kill corals and thereby further accelerating the coral-algal phase shift through this DOM-Disease-Algae-Microbes (DDAM) feedback loop (Barott & Rohwer, 2012; Dinsdale & Rohwer, 2011).

Despite its recognized importance for the functioning of marine ecosystems the makeup of DOM remained largely elusive due to methodological constraints that are just now being overcome. Advances in the application of both mass spectrometry and chromatography in combination with improved downstream analysis allow for increasing chemical insight into this complex mixture (Riedel & Dittmar, 2014; Zark, Christoffers & Dittmar, 2017; Zark & Dittmar, 2018). Only a small fraction (often 1%) of these DOM components can be identified directly *via* database matching (Petras et al., 2017). However, tandem mass spectrometry (LC-MS/MS) and the subsequent application of molecular networking and annotation propagation (Nothias et al., 2020; Wang et al., 2016; Ernst et al., 2019) have proven to be a powerful tool to increasingly characterize this highly complex pool of molecules (Petras et al., 2017).

First successful applications of this novel approach in reef environments revealed significant differences in the molecular composition of DOM released by various benthic primary producers (*i.e.,* exometabolomes) (Nelson, Wegley Kelly & Haas, 2023; Weber et al., 2020; Wegley Kelly, Haas & Nelson, 2018; Wegley Kelly et al., 2022) and how this composition changes under heat-stress (Sparagon et al., 2024). To better understand the role of DOM in reef degradation and identify potential strategies to mitigate the resulting feedback loops, it is indispensable to continue the molecular characterization of coral and algal DOM by tracking the decline of taxon-specific DOM components and linking their uptake to the growth of microbial communities. Therefore, we (1) characterized the exometabolomes released by a mixed community of corals and the macroalga *Dictyota sp.*, turf algae, as well as mixtures of coral-turf algae and coral-*Dictyota*. Subsequently, we (2) identified changes in exometabolome composition after 28 h of processing by an ambient bacterioplankton community, and (3) linked these compositional changes to bacterioplankton growth parameters. Using an untargeted metabolomics approach of LC-MS/MS allowed us to pinpoint exometabolome features from benthic primary producers, track their decline during microbial processing, and employ *in silico* predictions of compound classes.

## Materials and Methods

All benthic primary producers (BPP) used in this study were collected from the reef in front of the CARMABI research station at Piscadera bay (12°7′15″N, 68°58′12″W) on the Southern Caribbean Island of Curaçao in November 2018. The experiment was conducted at the CARMABI research station under the annual research permit of the CARMABI foundation (2022/21467) distributed by the Curaçaoan Ministry of Health, Environment, and Nature (GMN).

A selection of six different coral species was collected representing the coral community in species composition and abundance on the shallow reef terrace at the collection side. Collected species were: *Colpophyllia natans*, *Porites astreoides*, *Favia fragum*, *Stephanocoenia michelinii*, *Siderastrea siderea*, and *Orbicella annularis*. Corals were collected between 5–10 m depth and left on a floating coral nursery at five m depth on the collection site for 7 days. During this recovery period corals were cleaned from visible algae and checked daily for signs of decreasing health (*e.g.*, discoloration and/or tissue loss). No signs of decreasing health were observed. Coral rubble densely covered with either turf algae (heterogeneous consortia of *Chlorophyta, Phaeophyta*, and *Rhodophyta* commonly including filamentous cyanobacteria; Connell, Foster & Airoldi, 2014; Fricke et al., 2011) or thalli of the brown alga *Dictyota* sp. were collected wearing a clean set of nitrile gloves, just prior to the experiment from the same location and similar depth as the corals. Both corals and algae were transferred to the wet lab facilities of the research station on the day of the experiment. After the experiment, corals were first placed back in the nursery, and after a few days, glued back with putty-PC-plumbing epoxy where the corals were initially collected.

### Experimental setup

**Figure 1:**
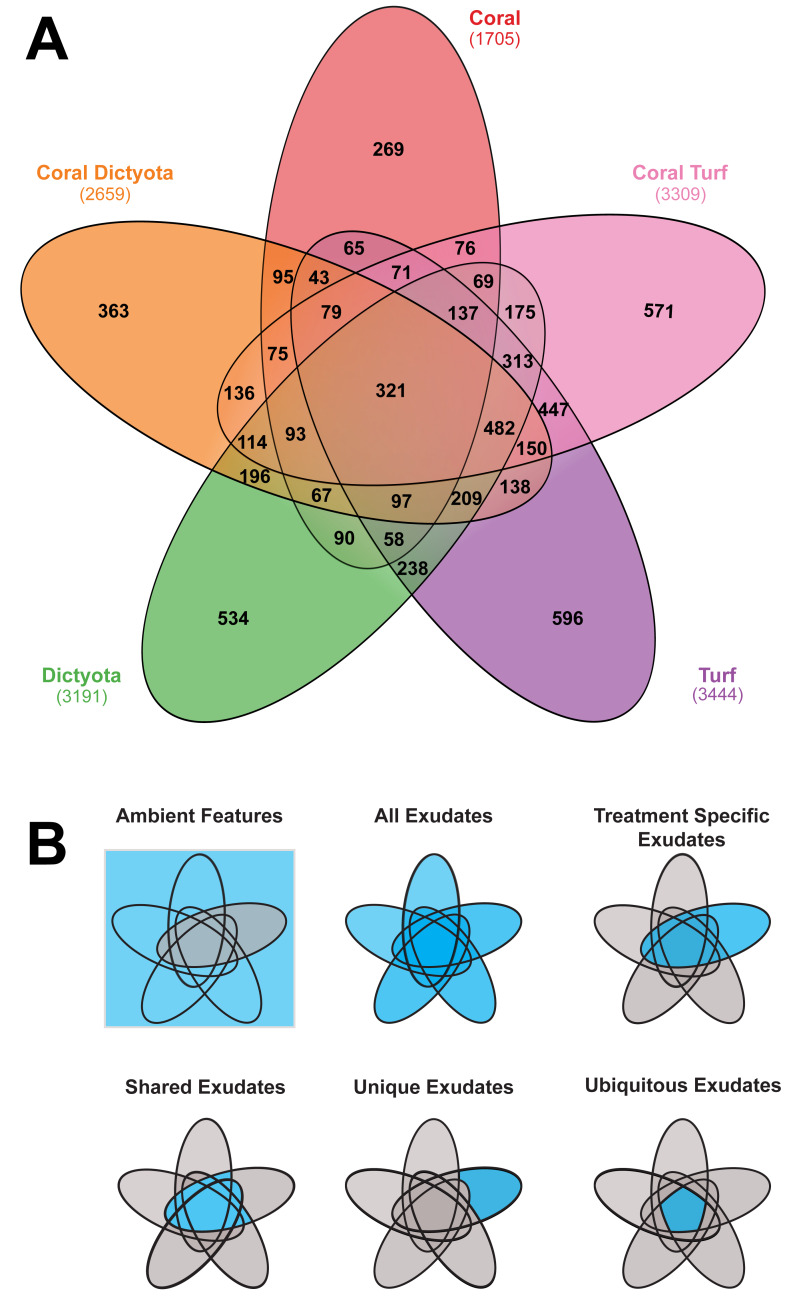
Characterization of exuded metabolites across treatments. (A) Venn diagram showing the number of unique and shared exometabolite features detected in each treatment (Coral,Coral_*Dictyota*, Coral_Turf, *Dictyota*, and Turf). Numbers within the overlapping regions indicate the number of features shared between those treatments. Numbers outside the overlapping regions indicate the number of features unique to each treatment. The total number of features detected in each treatment is shown in parentheses. (B) Conceptual illustration of the different categories of exometabolite features, for the treatment shown in blue under the “Treatment Specific Exudates” example. Ambient features are all features except the treatment specific exudates. Therfore a metabolite can be considered an exudate feature in one and an ambient feature in another treatment. All exudates are the treatment specific exudates of all treatments. Within the treatment specific exudates there is grouping of unique, shared or ubiquitous features.

Collected BPP were placed, while wearing clean nitrile gloves, in acid-washed (1.2 M HCl), opaque, high-density polyethylene (HDPE) containers filled with 20 L of filtered seawater (Sterivex 0.22 µm filter unit, EMD Millipore), pumped from the reef in front of the research station at 15 m depth. Percent cover of BPP in the containers ranged between 15% (turf algae) and 29% (*Dictyota*), to ensure a sufficiently high ratio of incubation water to BPP to avoid the accumulation of potentially harmful metabolic products or the depletion of essential components (Fig. 1). An identical container filled with filtered seawater only served as seawater control. Per exudation treatment (*i.e.,* coral, turf algae, *Dictyota* and seawater control) only one container was filled to avoid potential tank effects leading to compositional differences within replicate incubations per treatment and to ensure a single starting composition for subsequent dilution cultures to assess BPP-specific exudate processing by bacterioplankton. BPP and the seawater control were incubated in uncovered containers for 6 h (3 h before and after solar noon) to produce BPP-specific exudates. To prevent organic contamination no water exchange or aeration was applied. During these incubations, light intensities were similar to those at the nursery (verified with light and temperature loggers, Fig. S1). At the end of the incubations, BPP were removed using clean nitrile gloves and forceps and placed in holding tanks before being returned to the reef. Containers with BPP-enriched exudates and seawater controls were transferred to a temperature-controlled room (25 °C) and filtered (0.22 μm) into two-liter polycarbonate bottles to remove particles (including most bacteria). To determine the compound-specific removal of exudates in dilution cultures following procedures modified from Haas et al. (2011) we created three exudate treatments with 1,400 mL of coral-, *Dictyota*-, and turf algal-filtrate respectively and two mixed treatments with coral-filtrate (700 mL) and either *Dictyota*—(700 mL) or turf algal-filtrate (700 mL) added (Coral, *Dictyota*, Turf, Coral_*Dictyota*, and Coral_Turf, see Fig. S2). All mixtures were prepared in 2,000 mL polycarbonate bottles. A sixth treatment with filtered seawater (1,400 mL) served as control for the bioassays. Unfiltered seawater (600 mL) freshly collected from the nearby coral reef (<1 m above healthy coral community) was added to inoculate each bioassay with a natural bacterioplankton community. All six treatments were set up in quadruplicates of which one was sacrificed to characterize the initial DOM/exometabolome composition (see DOM extraction below). The remaining triplicates were incubated for 28 h in the dark at a constant temperature of 25 °C. Subsamples (one mL) to track bacterial cell growth (see Supplementary Methods: flow cytometry) and changes in bacterial size distribution (see Supplementary Methods: microscopy) were taken at five timepoints during the dark incubations (*t* = 0, 9, 18, 21, and 28 h). Flow cytometry and microscopy samples were fixed with 12 μL of glutaraldehyde and 16 μL paraformaldehyde, respectively, and stored at −80 °C until further analyses. After the final time point the remaining bioassay water was used to characterize the DOM/exometabolome composition after modification by the natural bacterioplankton community.

### Preparing and activation of PPL cartridges

The method described in Petras et al. (2017) was used for DOM extraction. Before the start of the field campaign Bond Elute Priority PolLutant (PPL) cartridges (Agilent 200 mg, 3 mL) had been pre-conditioned by rinsing each cartridge with 3 mL inhouse Bi-distilled methanol (MeOH), followed by overnight soaking with 3 mL MeOH. Subsequently, cartridges were rinsed twice with 3 mL ultra-pure water and once with 3 mL MeOH. After air drying, the cartridges were stored at room temperature until usage. During the field campaign, the PPL cartridges were re-activated before solid phase extraction by subsequently passing 3 ml of MeOH (BAKER ANALYZED, LC-MS grade) and two times acidified ultra-pure water of pH 2 (HCl, 0.001 M) through them.

### DOM extraction

Water samples for initial and final composition of DOM/exometabolomes were filtered through a 0.22 µm Sterivex filter (EDM Millipore; 1.2 L/hour). The filtrate was acidified to pH 2 with HCl (12 M) and slowly (0.25 L/hour) passed through the Bond Elute PPL cartridge to extract DOM components through solid phase extraction (Petras et al., 2017; Dittmar et al., 2008). After extraction, cartridges were first desalinated (rinsed three times with acidified ultra-pure water with pH 2) and then dried with N2 gas, before storage at −80 °C and transportation to The Netherlands to be analyzed.

### LC-MS/MS analysis

DOM was eluted from the cartridge with 2 mL LC-MS grade MeOH in pre-weighted combusted auto sampling vials, resulting in ∼1.5 mL extracts. Samples were split 50:50 and dried down overnight in a vacuum centrifuge. Dry weight of the DOM for both aliquot samples were determined and samples were stored at −20 °C till analysis. Samples were re-dissolved in 50 μL LC-MS grade MeOH and transferred into a combusted glass insert. 5 μL was inserted by a thermostatted auto-injector into an ultra-high performance liquid chromatography (UHPLC) system (Aglient 1290 Infinity I with a temperature-controlled column compartment) coupled to a Q-Exactive orbitrap mass spectrometer (Thermo Fisher Scientific, Bremen, Germany) equipped with ion max source with atmospheric pressure chemical ionization (APCI) probe (Thermo Fisher Scientific, USA). Chromatographic separation was achieved with a C18 core–shell column (Kinetex, 150 × 2 mm, 1.8 µm particle size, 100 A pore size, Phenomenex, Torrance, USA) and with a flowrate of 0.5 mL/min. Two solvents were used for the gradient program (solvent A: H2O + 0.1% formic acid (FA), solvent B: Acetonitrile (ACN) + 0.1% FA). After injection, the samples were eluted from 0 to 0.5 min in a mixture of 95% A and 5% B. Between 0.5 and 8 min the gradient changed linearly to a 50%–50% mixture of the two solvents. From 8 to 10 min the mixture changed—linearly—to a mix of 1% solvent A and 99% solvent B. The washout phase at 99% of solvent B followed for 7 min. Within one-minute (minute 17 to minute 18) the gradient was changed to the initial 95% A and 5% B mixture followed by a 7-minute (18–25 min) re-equilibration phase for the same mixture. Mass spectrometry measurements were performed in positive ion mode. Electrospray ionization (ESI) parameters were set to 52 L/min sheath gas flow, 14 L/min auxiliary gas flow, 0 L/min sweep gas flow and 400 °C auxiliary gas temperature. The spray voltage was set to 3.5 kV and the inlet capillary to 320 °C. 50 V S-lens level was applied (for detailed methods see Petras et al., 2017; Wegley Kelly et al., 2022). MS1 of DOM was analyzed within a mass range of 150–1,500 m/z with a resolution of 140,000. Data dependent MS2 data was obtained at a resolution of 17,000, in which the five most abundant masses in the mass spectrum—isotope peaks and ions with unassigned charged states excluded—were fragmented successively (stepped normalized collision energy of 20, 30, and 40%; isolation window 0.4 m/z and *z* = 1 as default charge state). Maximum injection time was set to 100 ms and automated gain control (AGC) target to 1.0E6 while the minimum AGC target was 5E4 and MS/MS experiments were triggered at the apex of peaks within 2–15 s from their first occurrence. Dynamic exclusion was set to 5 s (for detailed methods see Petras et al., 2017; Wegley Kelly et al., 2022).

### LC-MS/MS data processing

LC-MS/MS .raw data files were converted to mzML using MSConvert (Chambers et al., 2012) and feature picking and alignment was done using MZmine3 (Pluskal et al., 2010) (see Supplementary Methods). A total of 115 runs consisting of coral reef DOM samples (a subset of the run samples are the samples taken for this experiment), instrument blanks and procedure blanks (Milli-Q water processed alongside samples, filtered, and subjected to PPL extraction) from one analysis session were combined to increase MS^2^ spectra matching and identification. From all the 115 samples MZmine extracted 28,758 ion features for which MS/MS fragmentation spectra were available (from here on referred to as *features*) and calculated extracted ion chromatograms (XIC) for each sample based on peak area of the feature. The online Global Natural Products Social Molecular Networking (GNPS) environment (Wang et al., 2016) was used for feature based molecular networking (FBMN) (Nothias et al., 2020), ion identity networking (Schmid et al., 2021) and library search based on spectral similarity (see all settings in the Supplementary Methods). Spectra matching (cosine score > 0.7 and at least four matching peaks) resulted in 1,297 features that were annotated and assigned a library ID (*n* = 1,297, 4,5%). Subsequently ClassyFire (Djoumbou Feunang et al., 2016) assigned compound classes to these spectra matche structures. Within the GNPS environment the MolNetEnhancer tool (Ernst et al., 2019) was used to propagate ClassyFire compound class information across their molecular network (*n* = 3,386, 12%).

### Metabolomics data processing

From the 115 coral reef DOM samples that were run together on MZmine, 42 runs belong to this study (24 samples, 16 instrument blanks, two procedure blanks) and statistical analysis was performed using Rstudio (2023. 4.3.2). Features were flagged and removed if the maximum peak area in the blanks was higher than half of the mean peak area in the samples (13,819 features). By comparing the dataset before and after the MZmine gap filling step, the peak area background noise level was set on 4,090. Features that did not exceed this background noise level in at least two samples, were considered transient features and removed. This resulted in an additional removal of 1,673 features. The resulting dataset consisted of 13,266 features comprising both ambient features and benthic primary produced exudate features.

### Identification of ‘exudates’ and ‘metabolized features’

Exudate features were determined in the *T* = 0 samples. Features were tested for a two-fold increase in one of the treatments compared to the seawater control $log2 \left( \frac{sample}{control} \right) > 1$. In addition, the minimum peak area of the features was set to 1E6/60—about 4 times the peak area background threshold used during the transient feature removal—to exclude extreme small peaks.

**Figure 2:**
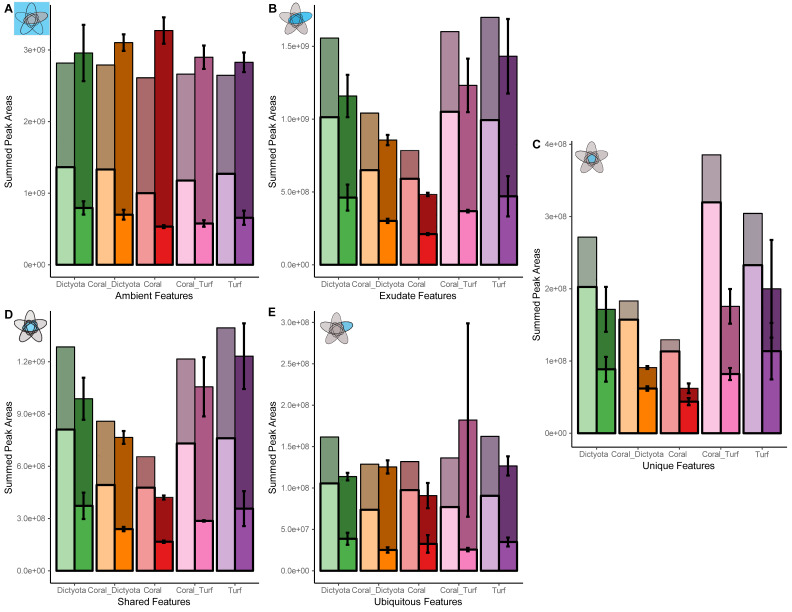
Changes in summed peak areas of exometabolite feature groups during incubation. Each panel (A–E) shows the summed peak areas at *T* = 0 (left, lighter bar) and *T* = 28 (right, darker bar) for each treatment. Each bar represents the summed peak area of all features in the respective group at that time point and is shown as the gray colored top bar. Each hatched bar shows a clear portion that represents the summed peak area of the subset of those features that decreased in peak area between *T* = 0 and *T* = 28. *T* = 0 represents a single replicate; while *T* = 28 values are the mean of three replicates, with error bars indicating standard deviations. Note that while the ubiquitous feature group contains the same features across all treatments, the number and identity of features within the exudate, ambient, unique, and shared groups vary by treatment (see Fig. 1). Tables 1 and 2 provide the ratios of ambient/exudate and shared/unique features, respectively. (A) Ambient features. (B) Exudate features. (C) Shared exudate features. (D) Unique exudate features. (E) Ubiquitous exudate features.

The features metabolized in the remineralization step were characterized *via* multiple steps. Firstly, a selection was made using a threshold of a 2-fold change of a feature between timepoint 0 and 28. $log2 \left( \frac{Tmax}{Tmin} \right) > 1$ or $log2 \left( \frac{Tmax}{Tmin} \right) < -1$. Features that passed this initial ‘metabolized feature finder’ test, were used in further analysis. To identify how groups of features within each treatment responded over time we selected features belonging to one of the groups (i) ambient, (ii) exudates, (ii) unique exudates, (iii) shared exudates and (iv) ubiquitous exudates (Fig. 1B) in each treatment and calculated the summed peak areas at both time points (Fig. 2). To specify how much of the (released) exometabolites were consumed in each treatment we repeated this calculation exclusively for all features that were decreasing over time. Statistical analysis applied to the comparisons were—chosen after verifying the assumptions of normality and homoscedasticity.

## Results

### Characterization of BPP-specific exometabolomes and exometabolome mixtures

At the end of the BPP incubations the overall dissolved organic carbon concentrations in all treatments ranged from 263 µmol L^−1^ in coral exudation treatments to 352 µmol L^−1^ in the *Dictyota* treatment (Table S1). A total of 13,266 ion-features were detected in the treatments, of which 6,901 features were present at similar concentrations in the seawater control and all primary producer treatments. Therefore these 6,901 features are part of the ambient features in all treatments. The other part of the ambient features is treatment specific (*i.e.,* a non-exuded feature in the respective treatment but exuded by one of the other treatments). The remaining 6,365 features were released as exudates by at least one of the respective primary producers (all exudate features). The molecular diversity (*i.e.,* number of exudate features) differed considerably among treatments, with coral exudates (1,705) containing approximately half the number of features compared to *Dictyota* (3,191), turf algae exudates (3,444), or the coral-algae mixtures (Coral-*Dictyota* = 2,659, Coral-Turf = 3,309) (Fig. 1/Table S2). Within the exudates, a fraction of features (13.6%–17.3%) was specific to certain treatments (unique exudate features) ranging from 269 unique exudate features in the coral treatment to 596 in the turf algae treatment. The majority of features was however shared between two or more treatments (*i.e.,* shared exudate features, Table 1) of which 321 were shared by all five treatments (*i.e.,* ubiquitous exudate features).

**Table 1:** Ambient to Exudate feature ratio. Ratios of ambient to exudate feature peak areas at the start (*T* = 0) and end (*T* = 28) of the incubation, for all features and for decreasing features only. ‘r’ represents the ratio of the number of ambient features to the number of exudate features.

	All			Decreasing		
Treatment	*T* = 0	*T* = 28	*r*	*T* = 0	*T* = 28	*r*
*Dictyota*	4.73	5.76	5.00	4.00	4.21	3.43
Coral_*Dictyota*	4.69	8.41	6.32	3.13	3.87	3.70
Coral	5.06	6.76	5.34	4.21	3.82	3.41
Coral_Turf	3.15	6.01	4.80	2.29	3.50	3.79
Turf	4.58	6.16	4.78	3.27	3.14	3.05

The ratio of the mean summed XICs of exudate to ambient features revealed treatment-specific differences in the relative contribution of exudate features. In coral treatments this ratio was about $ \frac{1}{3} $ compared to $ \frac{1}{2} $ in algal treatments suggesting a relatively higher contribution to the DOM pool by algae than by corals (Table 2). The exudate-ambient ratio of the coral-algal mixtures was in between the ratios of the two respective source exudates.

**Table 2:** Ratios of shared to unique feature peak areas at the start (*T* = 0) and end (*T* = 28) of the incubation, for all features and for decreasing features only. ‘n’ represents the ratio of the number of shared features to the number of unique features.

	All			Decreasing		
Treatment	*T* = 0	*T* = 28	*n*	*T* = 0	*T* = 28	*n*
**Dictyota**	1.81	2.55	3.16	1.35	1.72	1.67
**Coral_Dictyota**	2.68	3.62	3.99	2.05	2.32	1.96
**Coral**	3.33	6.77	6.78	1.69	2.53	2.44
**Coral_Turf**	1.66	2.35	3.01	1.12	1.57	1.78
**Turf**	1.56	1.97	2.85	1.28	1.40	1.59

### Changes in BPP-specific exometabolomes after processing by bacterioplankton

We investigated metabolites that decreased in the incubation experiments and interpreted this as evidence of drawdown by bacterioplankton remineralization. Here we considered features with a 2-fold decrease in peak area as metabolized in that respective treatment. The diversity of metabolized features was highest in the *Dictyota* treatment (4,406 features) followed by Coral_Turf (4,146), Turf (3,950), Coral_*Dictyota* (3,770), and Coral (3,126 features). While *Dictyota* had the highest variety of decreasing features the overall mean decrease in peak area was highest in Coral_Turf.

Although a higher number of ambient features was metabolized in each treatment, the average magnitude of decrease in exudate features (53–65%; Fig. 2B) was more pronounced than in ambient features (42–51%; Fig. 2A, analysis of variance (ANOVA), *p* < 0.001). This preferential use of exudate features is also reflected in the lower exudate-ambient feature ratio in decreasing features compared to all features in all treatments (Fig. 2/Table 2).

Among exudate features, on average, a higher proportion of unique exudate features are used (54–76%), compared to shared exudate features (43–48%) or ubiquitous exudate features (32–40%) (Figs. 2C, 2E/Table S2), indicating that microbes more frequently consume metabolites that are uniquely exuded. Unique exudate features further show the highest share of decreasing features in terms of summed peak area (*i.e.,* peak area of decreasing unique decreasing features/total peak area of unique features; 74–87%; Fig. 2D/Table S2). The magnitude of the change, the mean decrease in summed peak area was similar across feature categories (−51% to −74%; *t*-test, *p* = 0.33 and *p* = 0.42). This similarity suggest that, once consumption occurs, the extend of metabolite depletion is comparable between unique, shared, and ubiquitous exuded features. Taken together, these patterns point to a higher targeting of unique metabolites by microbial assemblages without implying differences in depletion intensity.

As they comprise identical features, the ubiquitous released exudates are directly comparable between the treatments. Between 104 and 129 of those ubiquitous features which made up between 55%–74% of the peak area were noticeably decreasing (Table S2), but only 30 features were decreasing in all five treatments. Even in the ubiquitously released exudates, each treatment had a unique breakdown signature (Fig. S2) consuming between 61%–66% of the ubiquitous feature summed peak area (Table S2).

**Figure 3:**
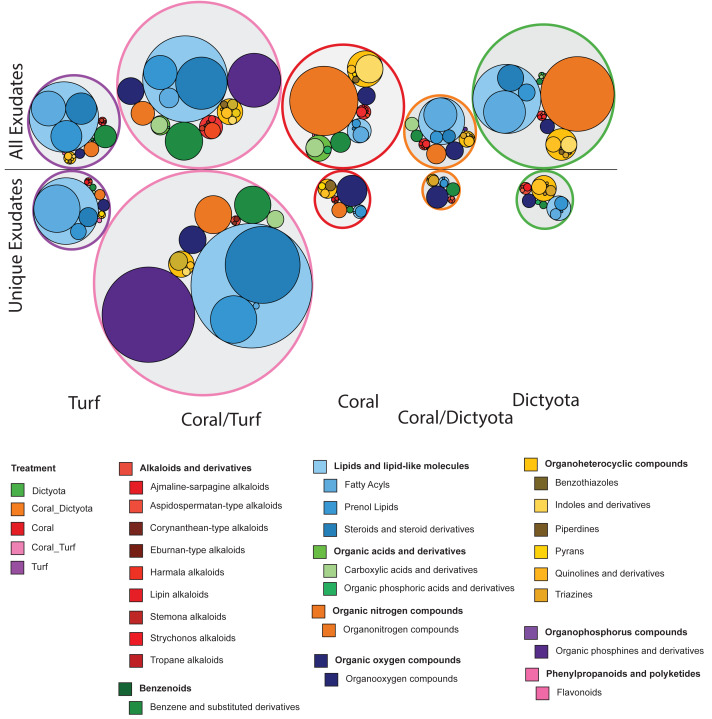
Relative summed decrease in peak area for ClassyFire chemical classes. The top row shows the relative summed *absolute* decrease in peak area for all decreasing exometabolite features assigned to each ClassyFire class within each treatment. The bottom row shows the same metric, but only for *unique* decreasing exudate features. For each treatment and chemical class, the absolute values of peak area decreases for all features within that class were summed. Circle sizes within each row are scaled relative to the maximum summed decrease within that row, meaning that circle sizes are not directly comparable between the top and bottom rows. Each color represents a different ClassyFire chemical class.

**Figure 4:**
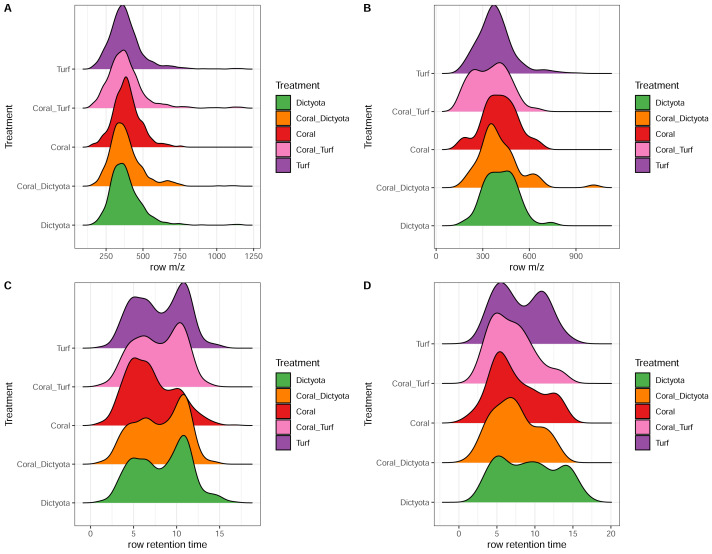
Distribution of *m/z* ratios and retention times of decreasing exometabolite features. (A) Distribution of *m/z* ratios for treatment-specific decreasing features. (B) Distribution of*m/z* ratios for unique decreasing exudate features (subset of A). (C) Distribution of retention times for treatment-specific decreasing features. (D) Distribution of retention times for unique decreasing exudate features (subset of C). The area under each curve represents the relative frequency of features.

Based on spectral properties 1,181 of the 13,266 features within this experiment (8.9%) were assigned a chemical Superclass and Class (Djoumbou Feunang et al., 2016). An overview of the number of features that made up the respective compound classes in each treatment can be found as Table S3. For each compound class in each treatment, we compared the decrease in summed peak area to both the initial summed peak area and the proportion of the initial summed peak area that was lost. This analysis revealed that the amount of peak area removed depends on the initial peak area (*i.e.,* inferred concentration) of the feature, yet this relationship is not strictly linear but differs between chemical classes (Fig. S4). Decreasing features in each treatment were assigned to a Class and Superclass and weighted by the summed absolute decrease in peak area (Fig. 3). This revealed clear differences in which compound classes were preferentially metabolized by the respective microbial community in each treatment. Figure 4 shows the distribution of the m/z ratios and retention times of the decreasing (unique) exudates features. With reverse phase chromatography polar features tend to elute fist followed by the more non-polar features, retention times are tied to the chosen gradient program (see methods). A pronounced consumption of lipid and lipid-like molecules could be observed in all treatments containing algal exudates, reflecting the decrease of the non- or a-polar signal at the longer retention time as shown Fig. 4C. The top of this mode is around 11–12 min, where the mobile phase consists of 99% acetonitrile. Within the ‘lipid and lipid like molecules’ superclass there is further differentiation with a more pronounced decrease of steroids and steroid derivatives in the Coral_*Dictyota* treatment, while in the other algal treatments fatty acyls are predominantly consumed (Fig. 3).

Organophosphorus compounds were dominantly consumed in the mixed Coral_Turf treatment, whereas the microbial consumption of phenylpropanoids and polycetides was specific to the turf algae treatment (Fig. 3). Organic nitrogen compounds—which are shared exudate features between the coral and the *Dictyota* treatments—were also dominantly consumed in these treatments. Similarly, organooxygen compounds were primarily found in coral and coral mixture treatments and were consumed in a higher amount in these than in the algal only treatments. Benzenoids were consumed in the turf and coral, but not the macroalgal treatments.

### Microbial growth and cell size in bioassay

**Figure 5:**
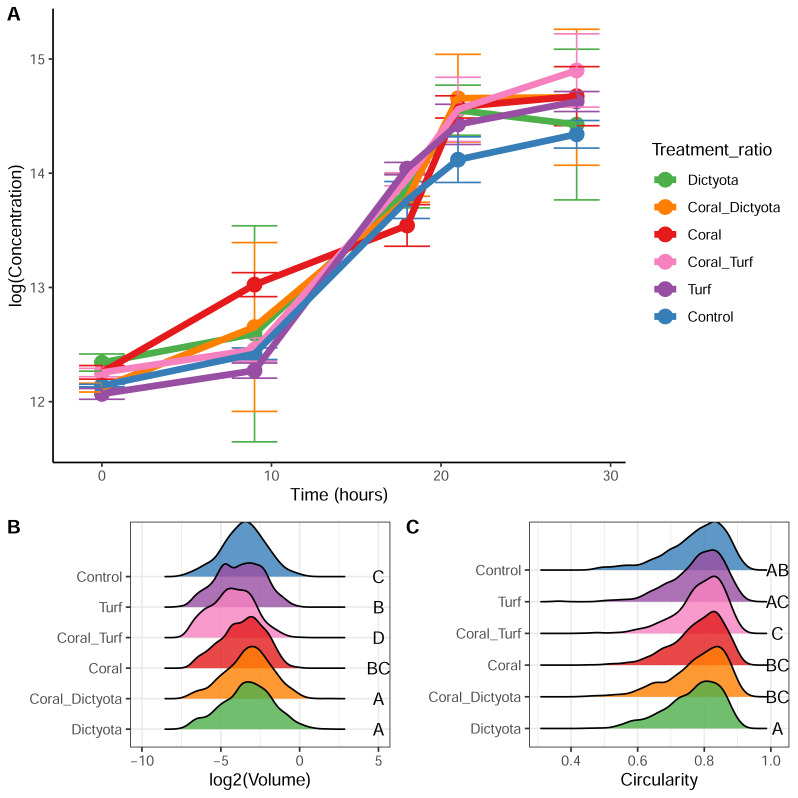
Microbial growth, cell volume, and circularity across treatments. (A) Microbial growth curves over 28 h in each treatment, measured using flow cytometry (FCM). Most treatments, with the exception of the Coral treatment, exhibit a lag phase during the first 9 h, followed by an exponential growth phase. The data suggest the onset of the stationary phase between 21 and 28 h. Error bars represent standard deviations of triplicate measurements. (B) Distribution of cell volumes at T=21 h, during the exponential growth phase. Different letters indicate statistically significant differences (ANOVA followed by TukeyHSD *post-hoc* test, *p* < 0.05). The Coral_Turf treatment exhibited significantly smaller cell volumes compared to the other treatments, while the *Dictyota* and Coral_*Dictyota* treatments yielded the largest mean cell volumes. (C) Distribution of cell circularity at T=21 h. Higher circularity values indicate more spherical (cocci) shaped bacteria, while lower circularity values suggest more elongated (vibrio or rod) shaped bacteria. Different letters indicate statistically significant differences (Kruskal–Wallis test followed by Dunn’s *post-hoc* test, *p* < 0.05).

During the 28-hour remineralization on the different exometabolites, the microbial population in all bioassays followed an exponential growth, and after 21 h the data suggest a transition into the stationary phase (Fig. 5A and Fig. S5). Maximum concentrations varied between 1.4E6–4.1E6 cells/mL, and growth rates in each bottle were not significantly different between treatments during the exponential phase (9h–21h) (ANOVA, *p* = 0.316) ranging from 0.098 ± 0.021 cells mL^−1^ h^−1^ (mean ± standard deviation (SD)) in the coral treatment, to 0.143 + 0.01 (Control), 0.157 ± 0.06 (Coral_*Dictyota*), 0.158 ± 0.09 (*Dictyota*), 0.173 ± 0.03 (Coral_Turf), and 0.184 ± 0.01 cells mL^−1^ h^−1^ in the Turf treatment.

Based on the growth curves, the 21-hour time point was chosen for microbial cell size analysis. Kruskal–Wallis test showed significant differences in mean cell volume between the treatments (*p* < 0.001). The *Dictyota* treatment (0.180 ± 0.240 μm^3^, *n* = 934) and Coral_*Dictyota* treatment (0.171 ± 0.202 μm^3^, *n* = 1,456; *Dictyota vs.* Coral_*Dictyota* = n.s.) featured the largest microbial cells followed by the Control treatment (0.135 ± 0.150 μm^3^, *n* = 1,010), Coral Treatment (0.122 ± 0.113 μm^3^, *n* = 840; Control *vs.* Coral = n.s.), and Turf treatment (0.116 ± 0.120 μm^3^, *n* = 380; Coral *vs.* Turf = n.s.), and the smallest microbial cells were found in the Coral_Turf treatment (0.08 ± 0.09 μm^3^, *n* = 1,040) which was the only treatment with significantly smaller microbes than all other treatments (Dunn’s test, *p* < 0.05).

Circularity of the cells was measured and compared between treatments. The mean circularity of microbial cells was lowest in the *Dictyota* treatment compared to all other treatments (Kruskal–Wallis and Dunn’s test, *p* < 0.01; Fig. 5C). In contrast, the other treatments showed no significant differences between them but was highest in the Coral_Turf treatment (0.788 ± 0.07, *n* = 1,040).

To assess the biomass in each dilution culture, the mean biovolume was multiplied by the concentration at T = 21 h for each replicate bottle. The Kruskal Wallis test showed no significant differences in the mean biomass among treatments (*p* = 0.131, Chi-square = 8.49, *n* = 16, DF = 5). However, Fig. S6 shows a clear trend indicating higher biomasses bacterioplankton community growing on *Dictyota* and the Coral_*Dictyota* treatment compared to Turf, Coral_Turf, and control treatments, with bacterioplankton communities of the coral treatment having intermediate biomass values.

## Discussion

To shed light on changes in the DOM pool and subsequent effects on bacterioplankton dynamics associated with coral-algal phase shifts on coral reefs we identified BPP-specific DOM components released by different BPP, tracked their decline during 28 h remineralization experiments with bacterioplankton communities, and recorded growth parameters of these communities.

### Characterization of exometabolites released by BPP

Comparable to previous studies, we found that coral reef BPP release distinct profiles of dissolved organic matter in which specific features released by the respective organisms can be identified within the complex mixture of DOM (Nelson et al., 2013; Wegley Kelly et al., 2022; Quinlan et al., 2018; Quinlan et al., 2019; Weber et al., 2022). Here we found more than 6000 distinct features that were released as exudates. While the proportion of treatment specific features were lower than in comparable studies from the central pacific (Wegley Kelly et al., 2022) there were still more than one third of the features specific to a respective treatment. Only about 5% were ubiquitously found in exudates of all BPP. Interestingly, there were some features that only appeared as unique features, or shared (361 and 144, respectively) in the mixture treatments. One possible explanation could be that exudates of the different treatments react together, creating features that were previously not present.

### Microbial changes of exometabolome

While a wide variety of features in all our treatments decreased noticeably during the incubation period, exudate features (*i.e.,* features that showed a two-fold increase in any exudation treatment compared to the seawater control), and specifically the features unique (*i.e.,* only occurring in one specific treatment) to a respective treatment, showed the highest percentage of decrease over the 28 h period (Fig. 2). This indicates a strong preference by the microbial community for these uniquely released metabolites as food source. We could further see that even within the ubiquitous released exudates there was a specific combination of features consumed that was characteristic for each treatment (Fig. S3). This suggests that not the availability of specific single compounds but rather the unique combination of compounds specific to each treatment is the main driver of microbial exometabolite consumption. This concept, that the addition of new resources selects for organisms that use those resources, has already been suggested in other systems. For instance, Docherty et al. (2006) showed clear changes in freshwater microbial communities after introducing DOM from new sources hypothesizing that a novel substrate (*i.e.,* different DOM composition) selects for a specific microbial community. Another reason for the changes in the microbial community may be that freshly released exudates contain more compounds that are structurally easier to access for microbes and therefore faster to metabolize, *i.e.,* more labile (Hansell, 2013). This would fall in line with other studies suggesting that benthic exudates are generally enriched in amino acids, vitamins and nucleosides which are considered labile compounds (Weber et al., 2022). Especially algal exudates were found to offer a larger amount of easily metabolizable sugars and other energy rich compounds (Nelson et al., 2013; Thobor et al., 2024; Wegley Kelly et al., 2022; Haas & Wild, 2010). The preference for unique exudate features could also be their higher initial abundance. Although we cannot infer actual concentration from peak areas, nor compare the peak area of two different features directly, we can derive relative abundance of one feature between samples or over time (America & Cordewener, 2008). Some features, that were exuded in abundance by a specific BPP, were also present in low concentrations as the background features in all the other treatments. These features were preferentially metabolized when actively exuded and present at high abundance. In contrast, their concentrations remained unchanged over time in all treatments when they occurred only as low-abundance background signals (Fig. S7). This is a known phenomenon and contributes to the difficult definition of recalcitrant DOM (Baltar et al., 2021). Regardless of whether it is a specific combination or a certain abundance of features, this study shows that a large variety of compounds are only metabolized when added fresh and/or abundantly as exudates. This result was expected, since microbial uptake is based on (but not limited to) diffusion and therefore concentration-dependent (Baltar et al., 2021). This allows microbes that specialize on metabolizing these specific compounds or specific combination of compounds to gain a competitive advantage, and, as a result, the molecular composition of exudates selects for a unique microbial community.

Besides investigating the general uptake patterns, we further used differences in LC column retention time as a proxy for polarity to compare groups of features between treatments, and used computational annotation tools to group features according to their putative molecular classes. The four treatments containing algal sourced DOM all show a dominant portion of consumed material to be lipids and lipid-like structures (Fig. 3; in light blue). Additionally, the retention time distribution of consumed compounds in Fig. 4C in these treatments show a distinct shape difference compared to the coral DOM treatment. The mode on the higher end of the retention time increased at a time in the gradient where lipid and lipid-like structures are typically eluted, suggesting that microbes growing on algal exudates preferentially metabolized less polar and more lipid-like compounds (Figs. 3 and 4). While these compound classes are initially more difficult to access than more polar compound classes (*e.g.*, sugars, amino acids) they are highly reduced and yield more energy. Such shifts in the community metabolism of microbes exposed to algal exudates have already been observed in previous studies that described increased abundances of copiotrophic microbial taxa when exposed to algal exudates in experiments (Nelson et al., 2013) or on algal-dominated reefs (Haas et al., 2016).

**Figure 6:**
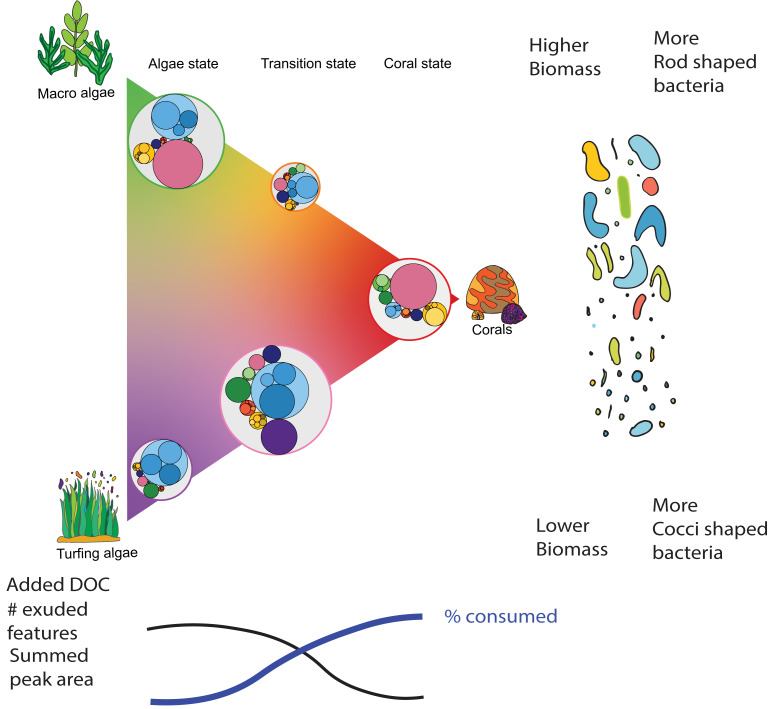
Conceptual model of microbial responses during coral-algal phase shifts. This figure illustrates the changes in dissolved organic carbon characteristics, microbial biomass, and bacterial cell morphology associated with shifts between coral-dominated (right) and algae-dominated (left) reef states. The triangle represents the gradient of benthic community composition, with macro algae and turf states on one side and coral on the other side. The sides of the triangle connnecting the algal with the coral states are representing transitions between these states. The relative size of circles within each state/treatment represents the summed peak area of consumed DOM of different chemical classes. The black line at the bottom represents the increase in added DOM, number of exuded features, and summed peak area from coral to algae states. The blue line shows the opposite trend, indicating the percentage of peak area consumed by bacteria, which is higher in the coral state. The bacterial shapes on the right illustrate the shift in dominant bacterial morphology and biomass, with coral-dominated states having lower biomass and more cocci-shaped bacteria, and algal-dominated states having higher biomass and more rod-shaped bacteria.

Both the coral as well as the macroalgae treatment resemble somewhat stable states in a reef system while the coral turf and coral macroalgae mixtures represent transitional states (Fig. 6). Turf algae, a heterogeneous consortia consisting of *Chlorophyta, Phaeophyta* and *Rhodophyta* commonly intermixed with filamentous cyanobacteria (Connell, Foster & Airoldi, 2014; Fricke et al., 2011; Steneck & Dethier, 1994) are generally the first benthic group to opportunistically colonize opened-up space during phase shifts (Diaz-Pulido & McCook, 2002) and can therefore also be considered a more transitional state during phase shifts.

Here, we show that these different states were also reflected by different compound classes that were preferentially metabolized in each state. The compound classes metabolized in the coral and macroalgae treatments were more similar to each other than to the mixture or turf treatments. The coral and the macroalgae treatments showed preferred microbial uptake of organonitrogen compounds and organoheterocyclic compounds.

The compound classes metabolized in the ‘transition’ treatments were also similar to each other. There was a pronounced decrease of benzenoids, including anthracenes, benzene and substituted derivatives, and prenol lipids in all treatments resembling transitional states. We could also see a higher decrease of organophosphorus compounds in these three treatments. This could potentially be facilitated *Flavobacteria* who are specialized organophosphorus compounds degraders (Singh & Walker, 2006) and have already been associated with algal-dominated reef sites (Haas et al., 2016).

### Microbial Response

Comparable to previous studies from different locations (*i.e.,* Virgin Islands, French Polynesia) and diverse experimental setups (*i.e.,* different incubation times, separate species, *etc.*), each of the BPP exudates elicited distinct responses in the ambient microbial community (Nelson et al., 2013; Weber et al., 2022; Haas et al., 2013; Silveira et al., 2019). While the different chemical mixtures supported microbial communities of similar abundances, the cells varied in sizes and shapes (Figs. 5B and 5C). Bacterioplankton communities growing on macroalgal DOM and coral-macroalgal DOM exhibited larger cell sizes (Fig. 5B) and higher biomass (Fig. S6). In contrast to previous studies (Silveira et al., 2019), the biomass of microbial communities was not increased when growing on turf algal exudates. This could possibly be explained by differences in the turf algal community composition between the studies, which can vary largely based on external parameters, such as collection depth or succession stage (Fricke et al., 2011). The relative abundance of eukaryotic and prokaryotic algal taxa is likely to result in considerable differences in the composition of DOM exuded by them.

Besides the size there were also differences in the shape of cells growing on macroalgal and coral treatments. Microbes growing exclusively on macroalgal exudates showed lower circularity compared to pure coral or coral-algal exudate mixtures (Fig. 5C). Macroalgal exudates may therefore select for rod-shaped microbes such as bacilli and vibrios, maximizing their surface-to-volume ratios and thereby nutrient uptake ability, whereas pure coral, and particularly coral-algal exudate mixtures may promote the growth of cocci or spherical shaped microbes featuring less favorable ratios for uptake (Young, 2006) (Fig. 6).

## Conclusion

Heterotrophic microbes on coral reefs can choose from a diverse suite of organic compounds present in the surrounding water. Here we can show for the first time that they prefer at least parts of the exudates added by the different BPP to the system. From these exudates the components unique to a respective dominant primary producer were the most favored substrate. This preference may be a mechanism selecting for different microbial community composition as specific taxa could gain a competitive advantage breaking down exudates unique to a specific BPP. Thus, the unique combination of organic compounds rather than specific single substances within the heterogeneous mixture of exudated seem to be driving changes in coral reef microbial communities during phase shifts.

##  Supplemental Information

10.7717/peerj.20748/supp-1 Supplemental Information 1 Supplementary data

10.7717/peerj.20748/supp-2 Supplemental Information 2 Supplementary tables and figures

## Additional Information and Declarations

### Field Study Permissions

The following information was supplied relating to field study approvals (*i.e.*, approving body and any reference numbers): All samples are taken from Curaçao, where they were collected under the collecting permits of the Curaçaoan Government provided to CARMABI (government reference: 2022/21467).

### Data Availability

The following information was supplied regarding data availability: The dissolved organic matter is available at CCMS: 10.25345/C5FT8DX4V. The LC-MS/MS data is available at MASSIVE: MassIVE MSV000097410,
https://massive.ucsd.edu/ProteoSAFe/dataset.jsp?task=5b48f73c412944329db314099452423b
The GNPS/FBNA workflow is available at Github:
https://gnps.ucsd.edu/ProteoSAFe/status.jsp?task=500b1aa4a504494bb6c9092068ed60d2. The MolNetEnhancer job is available at GNPS: https://gnps.ucsd.edu/ProteoSAFe/status.jsp?task=9a4af2f75c754d628a181a17c1b0b2e0. To start the R analysis first, a project was made:
https://github.com/NIOZ-DOM-Analysis/ProjectStarter. The metabolomic data was cleaned using:
https://github.com/NIOZ-DOM-Analysis/DataCleanup. The following analysis specifically for this project is available at Github:
https://github.com/NIOZ-DOM-Analysis/MCSMAC.
