# Supplementary material for: Preference for producer specific exudates shapes microbial communities in coral reefs
Source: PeerJ. 2026 Feb 9;14:e20748. doi: 10.7717/peerj.20748 (PMC12897362; doi:10.7717/peerj.20748)
Supplement: Supplemental Information 1 [file peerj-14-20748-s001.docx]

**Supplementary Methods**

**Incubations with benthic primary producers**

The amount of benthic primary producers (BPP) used for the production of exudates was determined based on percent cover in the incubation containers and ranged between 15% (turf algae) and 29% (*Dictyota*) (Supplementary Methods Fig. 1). This level of quantification based on project surface area ensured a sufficiently high ratio of incubation water to BPP to avoid the accumulation of potentially harmful metabolic products (e.g., oxygen, nitrite) or depletion of essential components (e.g., dissolved inorganic carbon) during the 6h light incubations. Since no BPP-specific release rates were quantified, more accurate measures of biomass (e.g., 3D surface area, dry weight, or organic carbon content) were not required.

**
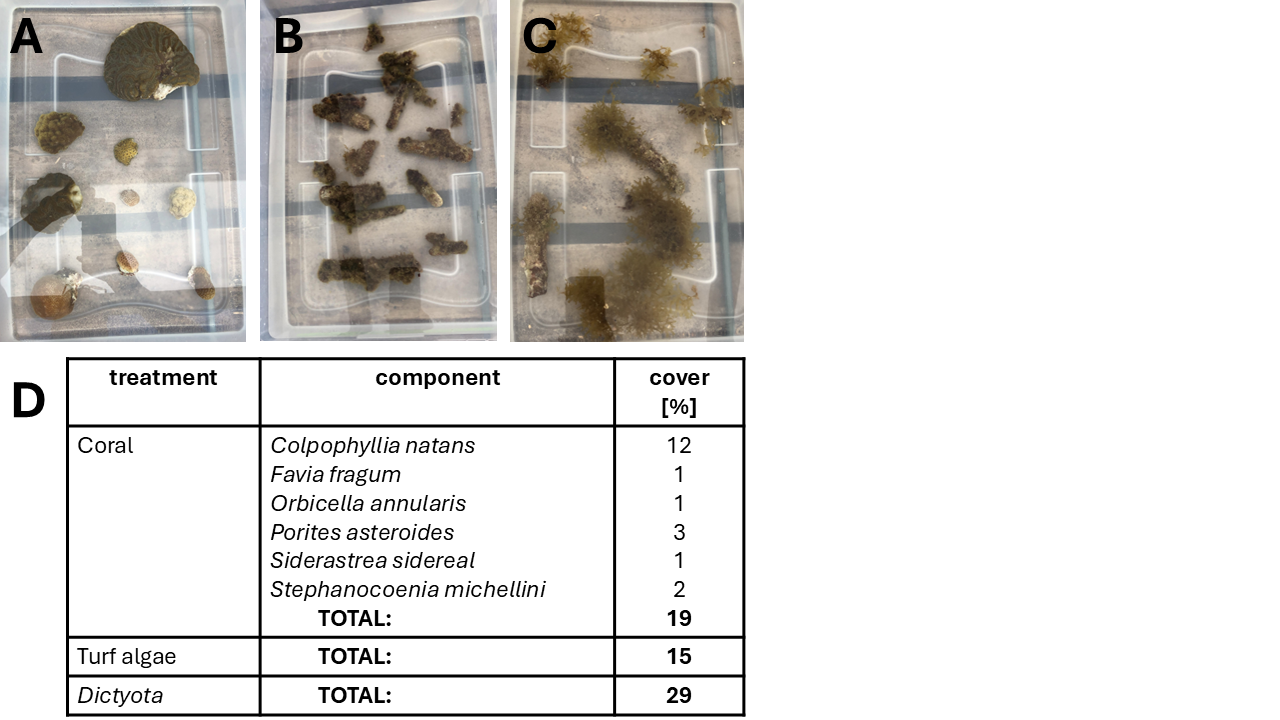
**

**Supplementary Methods Figure 1: Benthic primary producers used for the production for (A) coral, (B) turf algae, and (C) macroalgae exudates in 6 h light incubations. Percent cover of BPP based on project surface area are given in (D).**

**DOC analysis**

DOC samples (20 mL) were collected in pre-combusted (4 hours at 450°C), sample-rinsed glass vials closed with teflon-lined lids (10% HCl acid washed and sample-rinsed), acidified with 5 drops of 12 M HCl to a pH <2, and stored at 4°C until measurement. Concentrations of DOC were measured with high-temperature catalytic oxidation (TOC-L CPN, Shimadzu), calibrated with a standard curve of potassium hydrogen phthalate (0; 25; 50; 100; 200; 400 µmol C L^–^). Every sample was injected 5–7 times, resulting in an analytical variation of 2.1%. Measurement accuracy was tested by including consensus reference material (CRM; Batch 21, Lot: 04-21, DOC: 44.7 µmol L^−1^ ± 0.8 SD, D. A. Hansell, University of Miami) into every measurement run, which was on average 8% below the reported concentration.

**Microbial community**

**Flow cytometry**

Samples taken for flow cytometry (fixated with glutaraldehyde) were thawed at room temperature and added to freshly prepared and filtered TE buffer (0.5M EDTA + 1 M Tris; pH=8.2) to create a small range of dilutions. TE sample mixtures were stained with SYBR-Green and dark incubated appropriately for either virus or bacterial staining [1]. Samples were measured using the FACS Calibur with these certain settings. Measurement was done on medium flow for 1 minute with an average flowrate of 0.046 mL/min. Gating was done with the FCS Express 5.

Events within a gate were transformed to concentration (counts/mL). Since each sample was measured for 1 minute, concentration could be calculated by multiplying the counts by the dilution factor and divide by the flowrate.

**Microscopy**

As cell volumes and shape can be indicators of different microbial communities, the cell areas and Ferret’s maximum diameter were measured from a subset of cells from each treatment. Samples taken for microscopy (fixated with paraformaldehyde) were thawed at room temperature. Bacteria were stained with 25 μL DAPI [2] and collected on a 0.2μm polycarbonate filter (Whatmann). Filters were mounted on microscope slides and stored in the dark at -20°C. Slides were analyzed with epifluorescence microscopy using a Carl Zeiss AXIO Imager.M2 equipped with filter set 02, at 358 nm excitation and 463 nm emission wavelengths. Images were taken using the Zen Blue Pro software (Carl Zeiss), resulting in images of 2464 x 2056 pixels. The software was used to help determine the exposure time at intensity 30% to prevent bacteria from being overexposed. The UV light intensity was adjusted per sample for optimum contrast. Images were taken of multiple fields of view per slide. Filter area was not selected at random, but to ensure high numbers of equally distributed microbes in focus, and at the same time minimizing the amount of debris that could be falsely identified as cells by the software.

The Zen Blue Pro image analysis package (Carl Zeiss) was used to set up a semi-automated image analysis. Bacteria were detected by selecting only the high intensities of the blue layer of the 3 layers that make up the picture. The minimum blue light intensity was adjusted by hand based on the picture quality and background staining. The minimum area was set to 20, and minimum hole area to 1 and holes were filled. To separate the watersheds algorithm was used with the count of 3. Minimum circularity was set to 0.1. All images were checked and falsely identified microbes were removed. Clustering microbes not split by the watershed algorithm and thus detected as one object were split by hand. Multiple features of each detected object were measured but the Area and Feret Maximum are the only one used.

**Calculation of the microbial volume and weight.**

To calculate the volume of each microbe it was assumed that all microbes are rod shaped and thus geometrically a cylinder with two hemispherical ends. In a 2D picture the area is similar to the two half circles and a rectangle. Therefore, the volume (V) of a microbe can be calculated by the width (w) and length (l) of the microbe (equation 1). The width (w) was estimated from the area (A) and (l) shown by equation 2, where the feret maximum was used as a measure for length.

$$V=\frac{\pi}{4} w^{2}(l-\frac{w}{3})$$

$$w= \frac{2}{\pi-4}(\sqrt{l^{2}+\left( \pi-4 \right)A}-l$$

The volume (V) can be converted to dry weight (x) (equation 3), and with a linear relation the wet weight (z) was calculated (equation 4) [3]

$$\log(V)=1.72\log(x)-12.63$$

$$\log\left( z \right)=1.63\log\left( x \right)-2.0$$

By combining the flow cytometry data and the microbial size measured at T=21, the biomass waws calculated by multiplying the volume of each microbe by the concentration. Statistical analysis applied to the microbial data were chosen after verifying the assumptions of normality and homoscedasticity.

[DOC]

**LC-MS/MS data processing**

**MSConvert settings**

LC-MS/MS RAW data files were imported in MSConvert (ProteoWizard 3.0.19046 64-bit, [4]) to convert to open read mzXML files. Barcoding precision was set to 32-bit. Zlib compression was used as ‘write index’ and ‘TPP compatibility’. Filters applied were the Peak Picking filter with the Vendor Algorithm for MS Levels: 1-2 followed by the standard ‘titleMaker’ filter.

**MZmine3 settings**

Feature detection and alignment was done with MZmine3.2.8 [5]. The .mxXML files were imported using advanced import and both MS detectors were set as centroid and with noise levels of 1E5 and 1E3 respectively. ADAP chromatogram builder was set on MS level 1, with a minimum group size of 4 scans. Group intensity threshold was set to 1E5, and minimal highest intensity to 2E5. Scan to scan accuracy was set to 0.0015 m/z or 10 ppm. Local minimum feature resolver: MS/MS scan pairing was set up with a retention time tolerance of 0.15 min, and precursor tolerance levels were set to 0.1 m/z or 10 ppm. Dimension was set on retention time with a chromatic threshold of 85% and a minimum search range of 0.08. Minimum relative height was set to 0%, absolute height 2E5 and the minimum ratio of peak top/edge is 1.4. Peak duration ranged between 0-2 min with a minimum of 4 data points. 13C isotope filter was applied with a m/z tolerance of 0.001 m/z or 5 ppm. Retention time tolerance was set to 0.1 min. There was filtered on a monotonic shape and a minimum charge of 2. Representative isotope was set to most intense. Chromatograms were aligned with a m/z tolerance of 0.0015 m/z or 10 ppm and a retention time tolerance of 0.15 min. weight of m/z : RT was set to 3:1 and a mobility weight of 1. The feature list was filtered and included everything with minimum 2 features in an isotope pattern and with a MS2 scan. Gapfilling was done with the peak finder multithreaded module. Intensity tolerance was set to 10%; m/z tolerance of 0.0015 m/z or 10 ppm and retention time tolerance to 0.15 min with a minimum of 3 data points. Duplicate peaks were filtered with the ‘new average’ filter mode with mz tolerance of 0.001 or 5 ppm, and retention time tolerance of 0.05 min. Feature list was then renumbered, before being exported for GNPS-FBMN [6] workflow. Ion Identity networking was achieved by using the metaCorrelate module RT tolerance 0.5 min, min. height 0, intension correlation threshold 3E4, min samples in all 2 absolute, 0 in group, 60% min intensity overlap and gap-filled features are excluded. Correlation grouping was set up on 5 min data points with minimal 2 data points on edge, Pearson correlation was set up with a minimum feature shape correlation of 85%. After the metaCorrelate module, the ion identity module was used with the m/z tolerance of 0.0015 m/z or 5 ppm. Check was set on one feature with a minimum height of 0 and the ion identity library was set up using the positive MS mode, maximum charge of 2, maximum molecules/cluster 2. Adduct list: [M+H]+, [M+Na]+, [M+NH4]+. Selected modifications: [M-H2)]. Ion identities were added to the network with a m/z tolerance of 0.001 or 5 ppm with library in positive mode, max charge 2 and max molecules cluster 2. the adduct list: [M+H]+, [M+Na]+, [M+K]+, [M+NH4]+, [M+2H]2+, [M+H+Na]2+, [M+H+NH4]2+, [M-H+2Na]+, [M+Ca-H]+, and [M+Fe-H]+. Ion identities were added again with the same settings but added adducts were [M+H]+ and [M+NH4]+. Added modifications list: [M-H2O], [M-2H2O], [M-3H2O], [M-4H2O], [M-5H2O]. Ion identity network refinement was done by minimum size 2 and smaller networks were deleted that have less then 4 links. Ion identity networks were exported for GNPS/FBMN..

**GNPS** **settings**

The online platform GNPS: Global Natural Products Social Molecular Networking [7] was used to create a similarity network from the MS2 spectra (Feature Based Molecular Networking, [6] workflow version 28.2) using the MGF output from MZmine3. Precursor ion mass tolerance was 0.01 Da, as well as the fragment ion mass tolerance. To connect two nodes, which represent a feature, the cosine score threshold was set to 0.7 and a minimum of four fragment ion peaks had to match. Further, only the edges were kept if the connected nodes were in each others top 15 of most similar nodes. A maximum size of molecular family was set to 200. If a molecular family was over 200, the lowest scored edges are removed till the threshold of 200 was reached. Identification of the features is accomplished by matching MS2 data to the standard spectral libraries used by GNPS. Minimum of matching peaks was set to 4, score threshold was set to 0.7, and maximum analog mass difference was set to 200 Da. The in silico Dereplicator tool was also run [8]. Additional edges from the ion identity network were added. MolNetEnhancer [9] was used to propagate ClassyFire chemical ontology [10] on the network by using the in silico structure annotations from the GNPS Library Search. See data availability to find the perspective jobs.

**Data analysis**

The freely available software R version 4.3.2 (2023-10-31) in combination with RStudio (version 2023.9.1.494) was used. Installed packages are textclean, rmarkdown, knitr, kableExtra, tictoc, expss, vegan, stringi, psych, nortest, binom, epitools, car, ape, wesanderson, RColorBrewer, data.table, DescTools, broom, readxl, multcomp, summarytools, scales, reshape, reshape2, cluster, ggfortify, rfPermute, plyr, tidyverse, tibble, dplyr, svglite, dunn.test, UpSetR, gridExtra, grid, ggpubr, rstatix, ComplexUpset, cowplot, scatterplot3d, pdftools, png, magick, devtools, gridGraphics.

**Data availability:**

Raw data can be accessed through MASSIVE:
https://massive.ucsd.edu/ProteoSAFe/dataset.jsp?task=5b48f73c412944329db314099452423b

MassIVE MSV000097410 [ data available with the use of password MCSMAC till public ]

GNPS/FBNA workflow can be accessed here:

<https://gnps.ucsd.edu/ProteoSAFe/status.jsp?task=500b1aa4a504494bb6c9092068ed60d2>

The MolNetEnhancer job can be found at: <https://gnps.ucsd.edu/ProteoSAFe/status.jsp?task=9a4af2f75c754d628a181a17c1b0b2e0>.

To start the R analysis first a project was made using:
<https://github.com/NIOZ-DOM-Analysis/ProjectStarter>
and metabolomic data was cleaned using
<https://github.com/NIOZ-DOM-Analysis/DataCleanup/>

Following analysis specifically for this project is found here:

<https://github.com/NIOZ-DOM-Analysis/MCSMAC/>

**References**

1. Marie D, Brussaard CPD, Thyrhaug R, Bratbak G, Vaulot D. Enumeration of Marine Viruses in Culture and Natural Samples by Flow Cytometry. Applied and Environmental Microbiology. 1999;65: 45–52. doi:10.1128/AEM.65.1.45-52.1999

2. Kemp PF, Cole JJ, Sherr BF, Sherr EB. Handbook of Methods in Aquatic Microbial Ecology. CRC Press; 1993.

3. Simon M, Azam F. Protein content and protein synthesis rates of planktonic marine bacteria. Mar Ecol Prog Ser. 1989;51: 201–213. doi:10.3354/meps051201

4. Chambers MC, Maclean B, Burke R, Amodei D, Ruderman DL, Neumann S, et al. A Cross-platform Toolkit for Mass Spectrometry and Proteomics. Nat Biotechnol. 2012;30: 918–920. doi:10.1038/nbt.2377

5. Pluskal T, Castillo S, Villar-Briones A, Oresic M. MZmine 2: modular framework for processing, visualizing, and analyzing mass spectrometry-based molecular profile data. BMC Bioinformatics. 2010;11: 395. doi:10.1186/1471-2105-11-395

6. Nothias L-F, Petras D, Schmid R, Dührkop K, Rainer J, Sarvepalli A, et al. Feature-based molecular networking in the GNPS analysis environment. Nat Methods. 2020;17: 905–908. doi:10.1038/s41592-020-0933-6

7. Wang M, Carver JJ, Phelan VV, Sanchez LM, Garg N, Peng Y, et al. Sharing and community curation of mass spectrometry data with Global Natural Products Social Molecular Networking. Nat Biotechnol. 2016;34: 828–837. doi:10.1038/nbt.3597

8. Mohimani H, Gurevich A, Mikheenko A, Garg N, Nothias L-F, Ninomiya A, et al. Dereplication of peptidic natural products through database search of mass spectra. Nat Chem Biol. 2017;13: 30–37. doi:10.1038/nchembio.2219

9. Ernst M, Kang KB, Caraballo-Rodríguez AM, Nothias L-F, Wandy J, Chen C, et al. MolNetEnhancer: Enhanced Molecular Networks by Integrating Metabolome Mining and Annotation Tools. Metabolites. 2019;9: 144. doi:10.3390/metabo9070144

10. Djoumbou Feunang Y, Eisner R, Knox C, Chepelev L, Hastings J, Owen G, et al. ClassyFire: automated chemical classification with a comprehensive, computable taxonomy. J Cheminform. 2016;8: 61. doi:10.1186/s13321-016-0174-y
