# Supplementary material for: Preference for producer specific exudates shapes microbial communities in coral reefs
Source: PeerJ. 2026 Feb 9;14:e20748. doi: 10.7717/peerj.20748 (PMC12897362; doi:10.7717/peerj.20748)
Supplement: Supplemental Information 2 [file peerj-14-20748-s002.docx]

**Supplementary tables and figures**

**Supplementary Table 1: Dissolved Organic Carbon (DOC) and Total Nitrogen (TN) concentrations.** The table shows the average DOC and TN concentrations (µM) and their standard deviations (sd) of the samples (n=1) per treatment at the start of the incubation (Time Point 0). The n of DOC and TN represents the repeated measurements of the machine to assess the concentration of dissolved carbon and nitrogen in each sample.


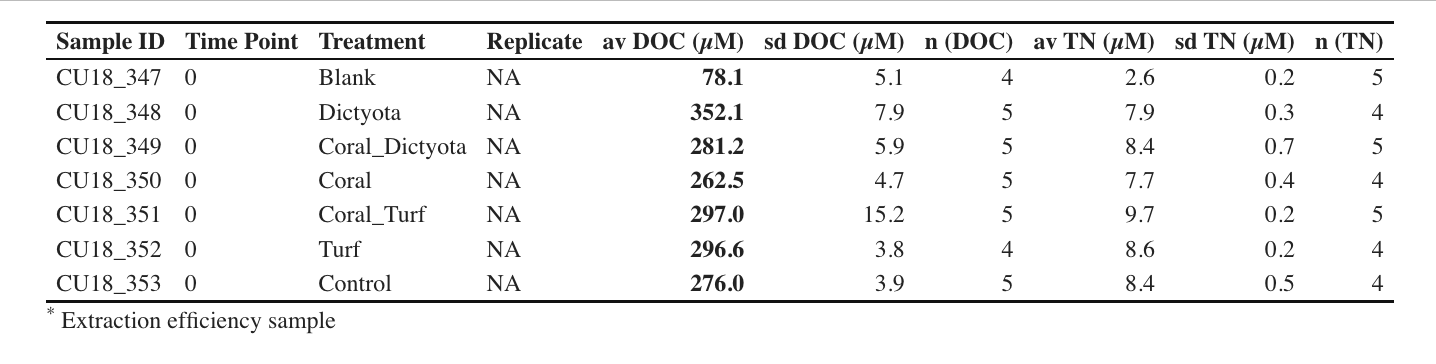


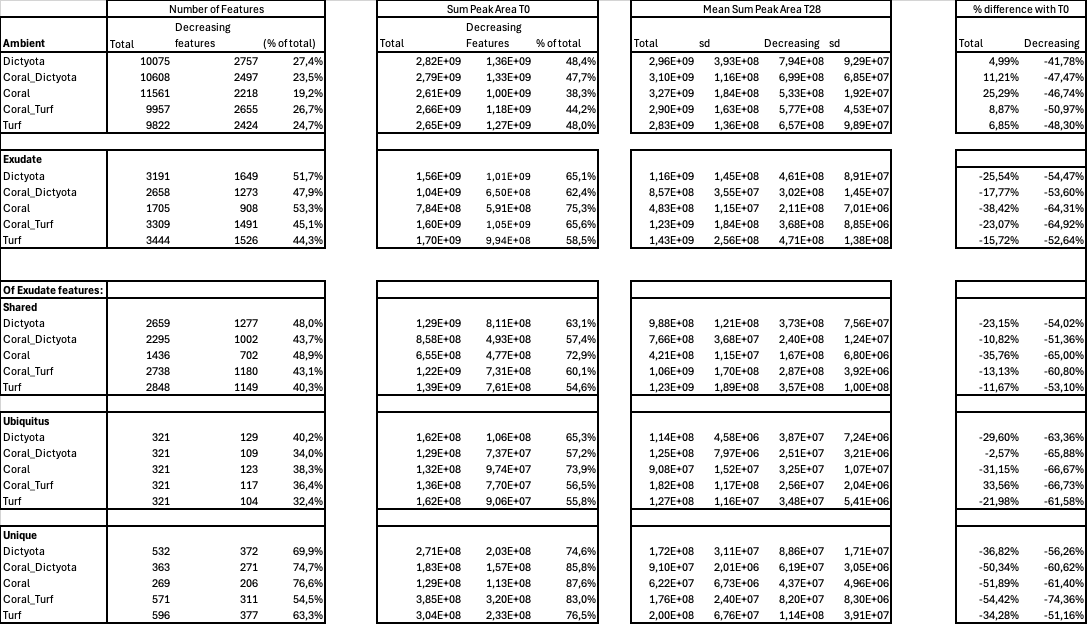
**Supplementary Table 2: Summary of exometabolite feature groups and their changes during incubation.** The table presents the total number of features, the number of decreasing features, and the percentage of decreasing features within each feature group (Ambient, Exudate, Shared, Ubiquitous, Unique) for each treatment. Additionally, it shows for both total and for the decreasing features 1) the summed peak area at T=0, 2) the mean summed peak area and the standard deviation of the summed peak area at T=28, and 3) the percentage difference in summed peak area between T=0 and T=28.

**Supplementary Table 3: Number of Decreasing Features per ClassyFire Class in Each Treatment.** This table shows the number of exometabolite features assigned to each ClassyFire chemical class that exhibited a decrease in peak area between T=0 and T=28 in each treatment (Coral, Coral_Dictyota, Coral_Turf, Dictyota, and Turf). "NA" indicates that no features belonging to that ClassyFire class were detected as decreasing in that particular treatment. This data is visualized in Figure 3.

| **CF_Superclass** | **CF_class** | Coral | Coral Dicytota | Coral Turf | Dictyota | Turf |
| --- | --- | --- | --- | --- | --- | --- |
| **Alkaloids and derivatives** | **Ajmaline-sarpagine alkaloids** | 2 | 1 | 1 | 1 | 3 |
| **Alkaloids and derivatives** | **Aspidofractine alkaloids** | NA | NA | NA | NA | 1 |
| **Alkaloids and derivatives** | **Aspidospermatan-type alkaloids** | NA | 2 | 1 | 2 | 1 |
| **Alkaloids and derivatives** | **Corynanthean-type alkaloids** | 1 | 1 | 1 | NA | 1 |
| **Alkaloids and derivatives** | **Eburnan-type alkaloids** | 2 | NA | 2 | NA | 1 |
| **Alkaloids and derivatives** | **Harmala alkaloids** | 1 | 1 | 2 | NA | NA |
| **Alkaloids and derivatives** | **Lupin alkaloids** | 1 | 1 | 1 | 1 | 1 |
| **Alkaloids and derivatives** | **Stemona alkaloids** | NA | 2 | 1 | 1 | 2 |
| **Alkaloids and derivatives** | **Strychnos alkaloids** | 1 | 1 | NA | 1 | NA |
| **Alkaloids and derivatives** | **Tropane alkaloids** | 1 | NA | NA | 1 | NA |
| **Benzenoids** | **Benzene and substituted derivatives** | 4 | 6 | 10 | 7 | 7 |
| **Lipids and lipid-like molecules** | **Fatty Acyls** | 12 | 12 | 27 | 28 | 36 |
| **Lipids and lipid-like molecules** | **Prenol lipids** | 7 | 22 | 28 | 21 | 25 |
| **Lipids and lipid-like molecules** | **Steroids and steroid derivatives** | 4 | 23 | 38 | 27 | 41 |
| **Organic acids and derivatives** | **Carboxylic acids and derivatives** | 3 | 4 | 4 | 3 | 1 |
| **Organic acids and derivatives** | **Hydroxy acids and derivatives** | NA | NA | 1 | NA | 1 |
| **Organic acids and derivatives** | **Organic phosphoric acids and derivatives** | 1 | NA | NA | 1 | 1 |
| **Organic nitrogen compounds** | **Organonitrogen compounds** | 5 | 5 | 8 | 6 | 6 |
| **Organic oxygen compounds** | **Organooxygen compounds** | 8 | 13 | 15 | 13 | 10 |
| **Organoheterocyclic compounds** | **Benzazepines** | NA | NA | NA | NA | 1 |
| **Organoheterocyclic compounds** | **Benzodiazepines** | 1 | 1 | 1 | 2 | NA |
| **Organoheterocyclic compounds** | **Benzopyrans** | NA | NA | 2 | NA | 2 |
| **Organoheterocyclic compounds** | **Benzothiazoles** | NA | NA | 1 | NA | NA |
| **Organoheterocyclic compounds** | **Indoles and derivatives** | 12 | 1 | 3 | 9 | 2 |
| **Organoheterocyclic compounds** | **Isoindoles and derivatives** | NA | NA | NA | 1 | NA |
| **Organoheterocyclic compounds** | **Oxazinanes** | NA | 1 | NA | 1 | NA |
| **Organoheterocyclic compounds** | **Piperidines** | 3 | 3 | 3 | 6 | 1 |
| **Organoheterocyclic compounds** | **Pyrans** | 1 | NA | NA | NA | NA |
| **Organoheterocyclic compounds** | **Quinolidines** | NA | NA | NA | 1 | NA |
| **Organoheterocyclic compounds** | **Quinolines and derivatives** | 6 | 2 | 6 | 10 | 4 |
| **Organoheterocyclic compounds** | **Triazines** | NA | 1 | 1 | NA | NA |
| **Organophosphorus compounds** | **Organic phosphines and derivatives** | NA | 1 | 3 | NA | NA |
| **Phenylpropanoids and polyketides** | **Flavonoids** | 1 | NA | NA | NA | NA |
| **Phenylpropanoids and polyketides** | **Isoflavonoids** | NA | NA | NA | 1 | 1 |
|  | **no matches** | 831 | 1169 | 1331 | 1505 | 1377 |

**Supplementary Table 4: Number of Unique Decreasing Exudate Features per ClassyFire Class in Each Treatment.**This table shows the number of unique exometabolite features (i.e., those found only in one treatment) assigned to each ClassyFire chemical class that exhibited a decrease in peak area between T=0 and T=28. "NA" indicates that no uniquely exudated features that is decreasing in the group of features belonging to that ClassyFire class were detected in that particular treatment. This data contributes to the visualization in Figure 3.

| **CF_Superclass** | **CF_class** | **Coral** | **Coral Dictyota** | **Coral Turf** | **Dictyota** | **Turf** |
| --- | --- | --- | --- | --- | --- | --- |
| **Alkaloids and  derivatives** | **Ajmaline-sarpagine alkaloids** | 1 | NA | NA | NA | 2 |
| **Alkaloids and  derivatives** | **Aspidospermatan-type alkaloids** | NA | 2 | NA | 1 | NA |
| **Alkaloids and  derivatives** | **Corynanthean-type alkaloids** | NA | NA | 1 | NA | NA |
| **Alkaloids and  derivatives** | **Eburnan-type alkaloids** | 1 | NA | 2 | NA | 1 |
| **Alkaloids and  derivatives** | **Lupin alkaloids** | NA | NA | NA | NA | 1 |
| **Alkaloids and  derivatives** | **Stemona alkaloids** | NA | 1 | NA | 1 | 1 |
| **Alkaloids and  derivatives** | **Strychnos alkaloids** | 1 | NA | NA | 1 | NA |
| **Benzenoids** | **Benzene and substituted derivatives** | 1 | 3 | 4 | 1 | 2 |
| **Lipids and lipid-like molecules** | **Fatty Acyls** | 1 | NA | 3 | 4 | 16 |
| **Lipids and lipid-like molecules** | **Prenol lipids** | 3 | 1 | 5 | 3 | 6 |
| **Lipids and lipid-like molecules** | **Steroids and steroid derivatives** | NA | 1 | 3 | 1 | 7 |
| **Organic acids and  derivatives** | **Carboxylic acids and derivatives** | 1 | NA | 2 | NA | 1 |
| **Organic acids and  derivatives** | **Organic phosphoric acids and derivatives** | NA | NA | NA | 1 | NA |
| **Organic nitrogen  compounds** | **Organonitrogen compounds** | 1 | NA | 5 | NA | 1 |
| **Organic oxygen  compounds** | **Organooxygen compounds** | 4 | 5 | 7 | 3 | 6 |
| **Organoheterocyclic  compounds** | **Benzazepines** | NA | NA | NA | NA | 1 |
| **Organoheterocyclic  compounds** | **Benzothiazoles** | NA | NA | 1 | NA | NA |
| **Organoheterocyclic  compounds** | **Indoles and derivatives** | NA | NA | 1 | NA | NA |
| **Organoheterocyclic  compounds** | **Isoindoles and derivatives** | NA | NA | NA | 1 | NA |
| **Organoheterocyclic  compounds** | **Oxazinanes** | NA | 1 | NA | 1 | NA |
| **Organoheterocyclic  compounds** | **Piperidines** | 1 | 1 | NA | 1 | NA |
| **Organoheterocyclic  compounds** | **Pyrans** | 1 | NA | NA | NA | NA |
| **Organoheterocyclic  compounds** | **Quinolidines** | NA | NA | NA | 1 | NA |
| **Organoheterocyclic  compounds** | **Quinolines and derivatives** | 3 | NA | 2 | 3 | 1 |
| **Organophosphorus  compounds** | **Organic phosphines and derivatives** | NA | NA | 2 | NA | NA |
| **Phenylpropanoids  and polyketides** | **Isoflavonoids** | NA | NA | NA | NA | 1 |
|  | **no matches** | 187 | 256 | 273 | 349 | 330 |

**Supplementary Figure 1: Light intensity measurements during incubations and comparison with reef and external conditions.**This figure presents light intensity measurements from different locations and logger types. (A) Light intensity recorded by Logger 2 in the greenhouse (green) and on the reef (red) on two consecutive days (2016-11-22 and 2016-11-23). (B) Light intensity recorded by Logger 1 outside the greenhouse on the same two days. The comparable light intensities recorded by both loggers on 2016-11-22 indicate similar light conditions between the reef and the external environment during that period, suggesting that the experimental setup in the greenhouse provided comparable light conditions to the reef. Note the dip in logger signal on the first day, where the logger ended up in the shadow of a nearby building and later was moved into the sun. (C) Comparison of light intensity measurements between HOBO loggers (Logger 1 and Logger 2) and PAR loggers during the experiment. This panel demonstrates similar light patterns recorded by both logger types. (D) Comparison of light intensity recorded by Logger 1 and Logger 2 when placed side-by-side on the same day.

**Supplementary Figure 2: Experimental setup.** Schematic overview of the experimental setup. Each bottle was used to extract one metabolomics sample. During the 28 hour incubation period small subsamples (<1 ml) were taken from the bottles (n=3) to analyze the growth and shape of the microbial community change over time.

**
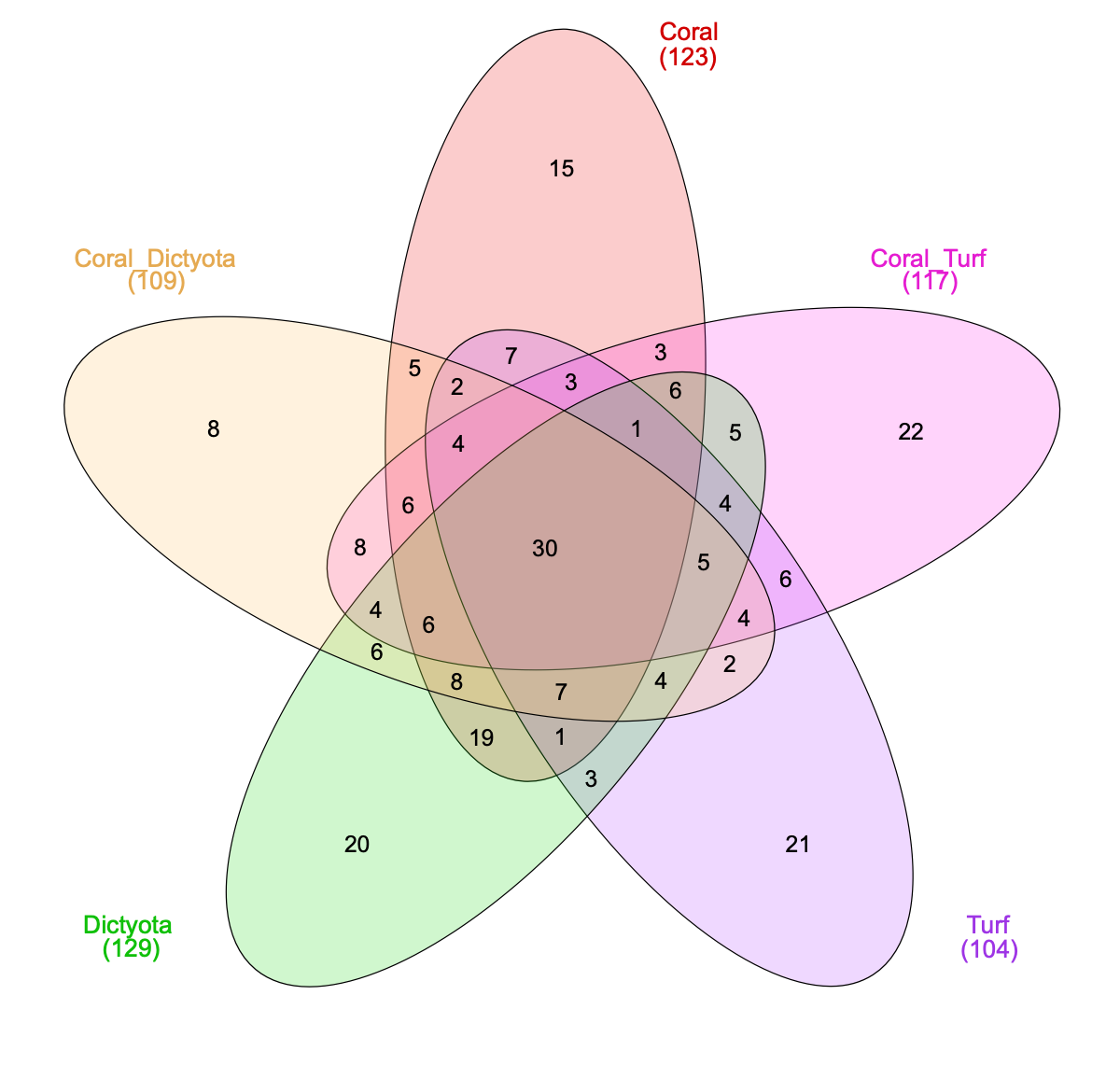
**

**Supplementary Figure 3: Overlap in decreasing ubiquitous exometabolite features across treatments.** This Venn diagram illustrates the number of ubiquitous exometabolite features that decreased in peak area during the 28-hour incubation period in each treatment. The numbers within each region indicate the number of features that decreased specifically in that combination of treatments. Note that while these features are ubiquitously released by all primary producers, their consumption patterns vary across treatments, highlighting the influence of treatment-specific factors on microbial metabolism.


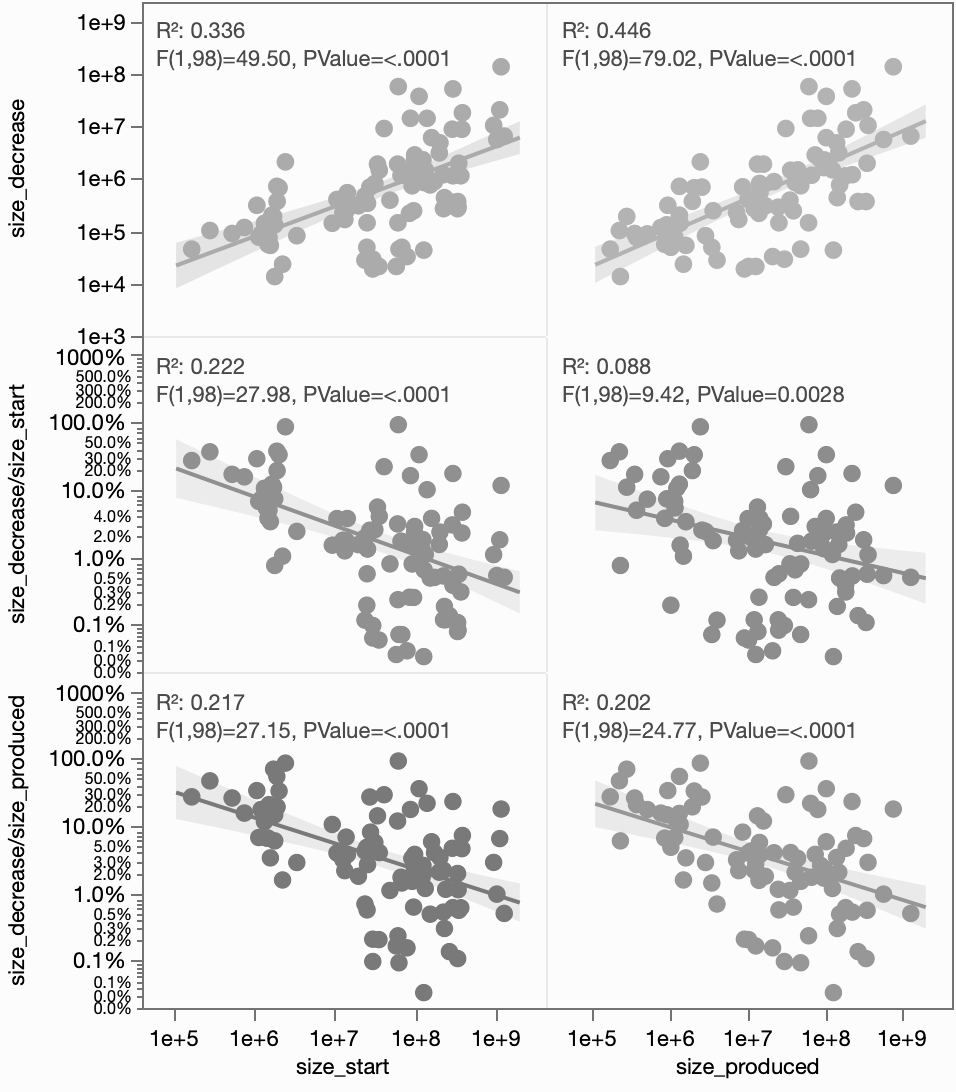


**Supplementary Figure 4: Relationship between initial peak area of decreasing exometabolite chemical classes and change in peak area during incubation.** This figure explores the relationship between the initial peak area (proxy for relative abundance) of decreasing exometabolite chemical classes and their subsequent change during the 28-hour incubation. Each dot represents a single chemical class observed in one of the treatments and that showed a decrease in peak area between T=0 and T=28 (as shown in figure 3). For each chemical class, we compared the decrease in summed peak area to both the initial summed peak area of exudate features and the proportion of the initial summed peak area that was lost. The top row shows the relationship between the initial peak area (size_start) and the absolute decrease in peak area (size_decrease). The middle row shows the relationship between the initial peak area (size_start) and the proportion of the initial peak area that decreased (size_decrease/size_start). The bottom row shows the relationship between the peak area produced by the BPP (size_produced, corrected by T=0 control samples) and the proportion of the produced peak area that decreased (size_decrease/size_produced). Each panel includes the R² value, F-statistic, degrees of freedom, and p-value from a linear regression analysis. The shaded areas represent 95% confidence intervals. The figure demonstrates that while larger initial peak areas generally correspond to larger absolute decreases, the relationship is not strictly linear, and the proportion of decrease varies considerably between chemical classes.


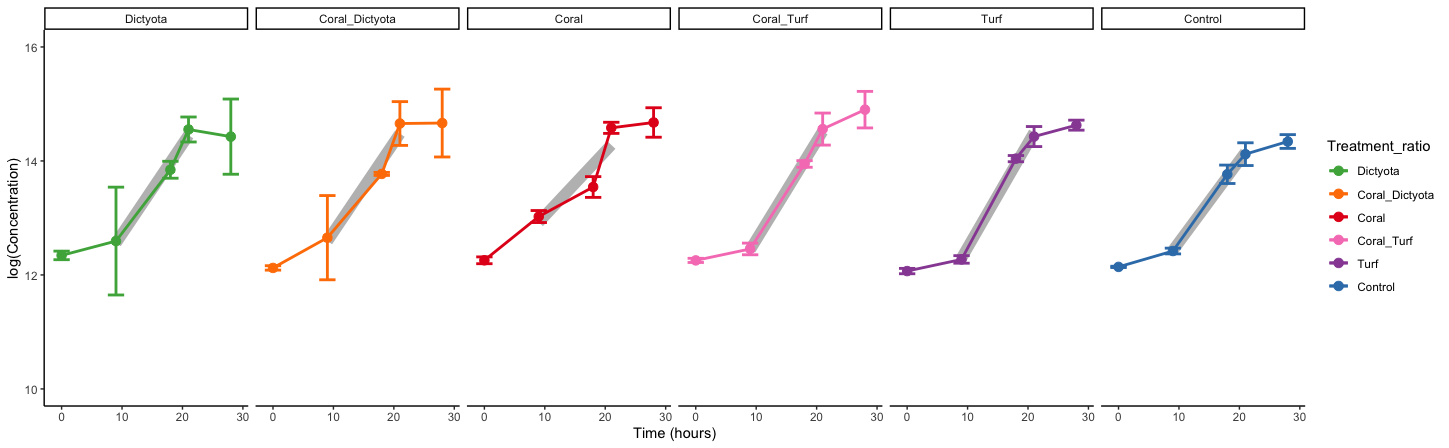


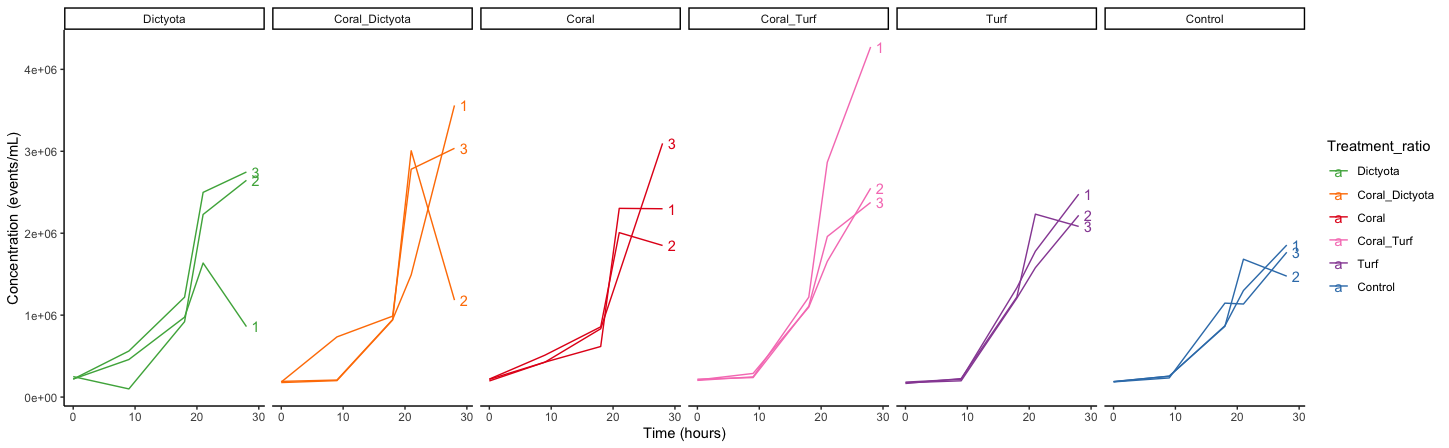


**Supplementary Figure 5: Microbial growth curves for each treatment.** Microbial cell concentrations over the 28-hour incubation period for each treatment are shown on a linear scale (top panel) with individual replicates plotted. The bottom panel shows the log-transformed mean cell concentrations with error bars representing standard deviations of triplicate measurements. The grey lines in the bottom panel represent linear regressions fitted to the mean log-transformed data between 9 and 21 hours, indicating the exponential growth phase.


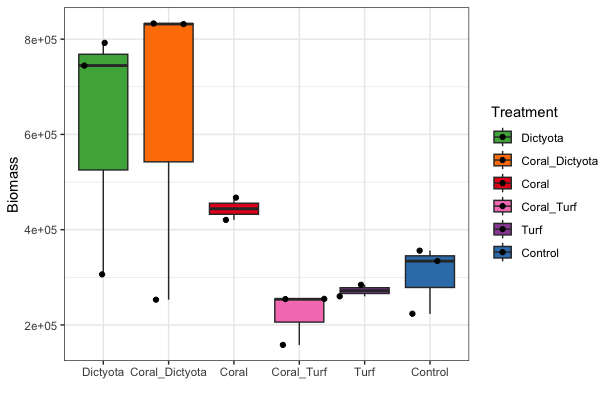


**Supplementary Figure 6: Bacterioplankton biomass across treatments after 21 hours of incubation.**

This figure presents boxplots showing the bacterioplankton biomass (calculated as the product of cell concentration and mean cell volume) measured after 21 hours of incubation in different treatments. Individual data points for each replicate are shown as black dots.

**Supplementary Figure 7: Summed peak areas of unique features across treatments at T=0 and T=28.** Each panel represents a different treatment, showing the summed peak areas of features unique to that treatment at the start (T=0) and end (T=28) of the incubation. Error bars represent standard deviations of triplicate measurements at T=28.
